# Reply to Ivy et al. Comment on “Khairul Zaman et al. Eco-Friendly Coagulant versus Industrially Used Coagulants: Identification of Their Coagulation Performance, Mechanism and Optimization in Water Treatment Process. *Int. J. Environ. Res. Public Health* 2021, *18*, 9164”

**DOI:** 10.3390/ijerph182312716

**Published:** 2021-12-02

**Authors:** Nadiah Khairul Zaman, Rosiah Rohani, Izzati Izni Yusoff, Muhammad Azraei Kamsol, Siti Aishah Basiron, Aina Izzati Abd. Rashid

**Affiliations:** 1 Department of Chemical & Process Engineering, Faculty of Engineering & Built Environment, Universiti Kebangsaan Malaysia, UKM, Bangi 43600, Malaysia; nadiahkz@gmail.com (N.K.Z.); nurul.izzati.izni@gmail.com (I.I.Y.); muhd.azraei24@gmail.com (M.A.K.); aishah@samb.com.my (S.A.B.); 2 Research Centre for Sustainable Process Technology, Faculty of Engineering & Built Environment, Universiti Kebangsaan Malaysia, UKM, Bangi 43600, Malaysia; 3 Makmal Pusat, Syarikat Air Melaka Berhad, Jalan Padang Keladi, Durian Tunggal, Melaka 76100, Malaysia; 4 Department of Civil Engineering, Faculty of Engineering & Built Environment, Universiti Kebangsaan Malaysia, UKM, Bangi 43600, Malaysia; ainaagibs@gmail.com

We appreciate very much the interest of Ivy et al. in our work [1] and for giving us the opportunity to clarify some points.

The relationship between aluminium (Al) concentration in drinking water and Alzheimer’s disease has been studied for several years [2,3]. One of the studies was carried out by Rondeau et al. (2000), the findings of which supported the hypothesis that Al in drinking water is a risk factor for Alzheimer’s disease based on 8 years of follow-up on their research [4]. The potential of having an increase in Al concentration in drinking water upon being treated using Al-based coagulant has also been presented in the guideline issued by World Health Organization (WHO) on Al in drinking water [5].

In view of this concern, our recently published manuscript aimed to compare the performances of Al-based coagulants and eco-friendly coagulant and to optimise their utilisation in coagulant dosing for various water sources. Investigation of the effect of applying different dosing parameters such as coagulant dosages, initial pH, and type of coagulants on turbidity and metal removal were specifically carried out. This lab-scale study is one step towards implementation at plant scale as it is a usual practice for optimising coagulant dosage and pH via jar testing. 

The optimisation performed in this study was carried out using Response Surface Methodology (RSM) and the summary of the obtained results shows that chitosan owes fewer sensitive responses (turbidity and residual metal) to the change in its input factors (dosage and pH), especially in acidic conditions, which suggested the beneficial role that its use has in non-critical dosage monitoring. Meanwhile, ACH was found to perform better than chitosan in removing turbidity and metals such as aluminium, manganese, and ferum only at pH > 7.4 with half dosage required. In summary, chitosan and ACH were seen to only perform equally at a different set of optimum conditions. The optimisation study offers precise selections of coagulants for a practical water treatment operation in ensuring that turbidity and metal can be removed to the allowable limit.

In the recently published work, issues related to the effect of utilising aluminium-based coagulant in drinking water treatment such as increasing water turbidity from aluminium hydroxide precipitates, reducing disinfection efficiency, and excessive headloss development in filters and distribution system have not been presented as suggested by Ivy et al. [6]. This research is still ongoing and will be published in the near future.
